# LAG-3, TIM-3 and VISTA Expression on Tumor-Infiltrating Lymphocytes in Oropharyngeal Squamous Cell Carcinoma—Potential Biomarkers for Targeted Therapy Concepts

**DOI:** 10.3390/ijms22010379

**Published:** 2020-12-31

**Authors:** Nora Wuerdemann, Katharina Pütz, Hans Eckel, Rishabh Jain, Claus Wittekindt, Christian U. Huebbers, Shachi J. Sharma, Christine Langer, Stefan Gattenlöhner, Reinhard Büttner, Ernst-Jan Speel, Malte Suchan, Steffen Wagner, Alexander Quaas, Jens P. Klussmann

**Affiliations:** 1 Department of Otorhinolaryngology, Head and Neck Surgery, University of Giessen, Klinikstrasse 33, 35392 Giessen, Germany; claus.wittekindt@klinikdo.de (C.W.); Shachi.Sharma@uk-koeln.de (S.J.S.); Christine.langer@hno-med.uni-giessen.de (C.L.); steffen.wagner@hno.med.uni-giessen.de (S.W.); 2 Department of Otorhinolaryngology, Head and Neck Surgery, Medical Faculty, University of Cologne, Kerpener Strasse 62, 50937 Cologne, Germany; hans.eckel@uk-koeln.de (H.E.); s0rijain@uni-bonn.de (R.J.); christian.huebbers@uk-koeln.de (C.U.H.); malte.suchan@uk-koeln.de (M.S.); jens.klussmann@uk-koeln.de (J.P.K.); 3 Center for Molecular Medicine Cologne (CMMC), Faculty of Medicine and University Hospital Cologne, University of Cologne, Robert-Koch-Str. 21, 50931 Cologne, Germany; 4 Institute of Pathology, University of Cologne, Kerpener Strasse 62, 50937 Cologne, Germany; katharina.puetz@uk-koeln.de (K.P.); Reinhard.buettner@uk-koeln.de (R.B.); alexander.quaas@uk-koeln.de (A.Q.); 5 Jean-Uhrmacher-Institute for Otorhinolaryngological Research, University of Cologne, Geibelstrasse 29–31, 50931 Cologne, Germany; 6 Institute of Pathology, University of Giessen, Langhansstrasse 10, 35392 Giessen, Germany; Stefan.gattenloehner@patho.med.uni-giessen.de; 7 Department of Pathology, GROW-School for Oncology and Developmental Biology, Maastricht University Medical Center, Universiteitssingel 40, 6229 ER Maastricht, The Netherlands; ernstjan.speel@mumc.nl

**Keywords:** oropharyngeal squamous cell carcinoma, human papillomavirus, LAG-3, TIM-3, VISTA, CD8-positive T-lymphocytes, tumor microenvironment

## Abstract

Tumor growth and survival requires a particularly effective immunosuppressant tumor microenvironment (TME) to escape destruction by the immune system. While immunosuppressive checkpoint markers like programmed cell death 1 ligand (PD-L1) are already being targeted in clinical practice, lymphocyte-activation-protein 3 (LAG-3), T-cell immunoglobulin and mucin-domain containing-3 (TIM-3) and V-domain Ig suppressor of T cell activation (VISTA) inhibitors are currently under investigation in clinical trials. Reliable findings on the expression status of those immune checkpoint inhibitors on tumor-infiltrating lymphocytes (TILs) in the TME of oropharyngeal squamous cell carcinoma (OPSCC) are lacking. This work aims to describe the expression of LAG-3, TIM-3, and VISTA expression in the TME of OPSCC. We created a tissue microarray of paraffin-embedded tumor tissue of 241 OPSCC. Expression of the immune checkpoint protein LAG-3, TIM-3, and VISTA in OPSCC was evaluated using immunohistochemistry and results were correlated with CD8+ T-cell inflammation and human papillomavirus (HPV)-status. 73 OPSCC stained positive for LAG-3 (31%; HPV+:44%; HPV-:26%, *p* = 0.006), 122 OPSCC stained positive for TIM-3 (51%; HPV+:70%; HPV-:44%, *p* < 0.001) and 168 OPSCC (70%; HPV+:75%; HPV-:68%, *p* = 0.313) for VISTA. CD8+ T-cells were significantly associated with LAG-3, TIM-3 and VISTA expression (*p* < 0.001, *p* < 0.001, *p =* 0.007). Immune checkpoint therapy targeting LAG-3, TIM-3, and/or VISTA could be a promising treatment strategy especially in HPV-related OPSCC. Future clinical trials investigating the efficacy of a checkpoint blockade in consideration of LAG-3, TIM-3, and VISTA expression are required.

## 1. Introduction

Immune checkpoints (ICP) are expressed in healthy tissue to prevent autoimmune disease and are often being altered by cancer cells to evade the host immune system [1]. Bypassing immune surveillance and immune response of tumor cells is controlled by the upregulation of co-inhibitory checkpoints and the delivery of inhibitory signals to T-cells. Tumors activate certain ICP, particularly against tumor-antigen specific T-cells, as a mechanism of immune resistance [2].

In recent years, ICP blockage has emerged in therapy of multiple cancer entities with encouraging results [3,4]. Whereas cytotoxic T lymphocyte-associated antigen 4 (CTLA-4) and programmed death 1 (PD-1) and ligand 1 (PD-L1) are the most extensively studied and targeted ICP receptors in treatment of multiple solid tumors, further next-generation ICP are within reach [5,6,7,8].

Lymphocyte-activation gene 3 (LAG-3) belongs to the immunoglobulin superfamily (IgSF) and is displayed particularly on activated immune cells e.g., several forms of T-lymphocytes (CD4+, CD8+, regulatory T-cells (Treg) [9,10]. There is an alternative splice variant of LAG-3 that leads to a soluble form (sLAG-3) with controversial biological functions of the protein [11]. LAG-3 binds with higher affinity than CD4 to major histocompatibility complex II (MHC II). This is supported by its gene sequence, which is 20% identical to CD4 [9]. The LAG-3/MHC II complex on CD4+ cells negatively modulates T-cell activity and enhances antigen self-tolerance when displayed on CD8+ cells. Persistent antigen exposure in the tumor microenvironment possibly results in maintenance of LAG-3 expression on inflammatory cells, which contributes to a state of exhaustion (e.g., impaired proliferation of T-cells and cytokine production) and can enhance anti-tumor T-cell response [12,13,14].

T-cell immunoglobulin and mucin-domain containing-3 (TIM-3) is expressed by a variety of immune cells including dendritic cells, macrophages, and T-cells and mediates its suppressive activity on immune cells via its ligands phosphatidylserine, CEACAM-1 and the widely expressed ligand galectin-9 [15,16,17,18,19]. TIM-3 is expressed on activated T-cells and its signaling on cytotoxic T-cells leads to an exhausted phenotype, characterized by a reduction in proliferation, decreased production of effector cytokines and apoptosis of effector T-cells [16]. Multiple studies have reported on the presence of TIM-3 tumor-infiltrating T-lymphocytes (TILs) in human tumors with various effects [20,21,22,23,24].

V-domain Ig suppressor of T cell activation (VISTA) shares homology with PD-L1 and is another ICP expressed on TILs and myeloid cells simultaneously functioning as ligand on antigen-presenting cells and as receptor in T-lymphocytes [25,26,27,28,29,30,31]. When upregulated, VISTA suppresses T-cell activation, proliferation, and cytokine production [32].

Numerous studies have demonstrated that the success of immunotherapy is often limited to a specific subgroup. This also applies to head and neck cancer (HNSCC) patients, where objective response rates are about 15% [33,34,35]. Oropharyngeal squamous cell carcinoma (OPSCC) displays a subgroup of HNSCC with increasing incidences [36,37,38]. Besides nicotine and alcohol, the development of OPSCC is caused by persistent infection with high-risk human papillomavirus (HPV), predominantly type 16 [39,40]. HPV-related OPSCC are preferentially located in lymphoid tissue of the head and neck (tonsil, base of tongue) and dysregulation of the immune system in their surroundings might play an important role in carcinogenesis. While most patients with HPV-related OPSCC are characterized by superior locoregional control and favorable outcome in comparison to patients with HPV-negative OPSCC [41], morbidity and post-treatment toxicity rates are still high in both subgroups.

Therefore, more effective and less toxic treatment strategies are urgently needed in this entity and new immune checkpoint inhibitor (ICI)-approaches might enable such personalized therapies in the future. Recent clinical trials are investigating the blockage of e. g. LAG-3 (Trials: NCT02061761; NCT01968109, NCT03538028, NCT03625323), TIM-3 (Trials: NCT03652077) or VISTA (Trial: NCT02671955) in multiple solid cancers, including HNSCC.

The expression profile of LAG-3, TIM-3, and VISTA on immune cells in OPSCC displays the basis for applying targeted therapies in the future. However, little is known to this point.

Therefore, we aimed to analyze the expression profile of targetable ICP like LAG-3, TIM-3, and VISTA in association with each other and according to HPV-status in a well-characterized, retrospective OPSCC patient cohort.

## 2. Results

### 2.1. Patient and Tumor Characteristics

Clinicopathological details of the OPSCC patient cohort are presented in Table 1. The median age of OPSCC patients was 60 years, whereas it was 60.6 in HPV-negative and 57.9 years in HPV-related OPSCC patients. Among the 241 cases, 63/241 (26%) patients were diagnosed with an HPV-related OPSCC (positive for high-risk HPV-DNA and p16^INK4a^ (p16) expression) and 177/241 (74%) with an HPV-negative OPSCC (Table 1). Patients with an HPV-related OPSCC were less frequently smokers and drinkers (each *p* < 0.001) and tumors were predominantly located in the tonsil region (*p* = 0.012) and associated with lymph node metastasis (*p* = 0.001). Patients with HPV-related OPSCC were more often treated with surgery initially in comparison to patients with HPV-negative OPSCC (*p* = 0.006) and patients with HPV-negative OPSCC developed recurrent disease more frequently (*p* < 0.001).

### 2.2. Expression Profile of LAG-3, TIM-3 and VISTA

**Figure 1:**
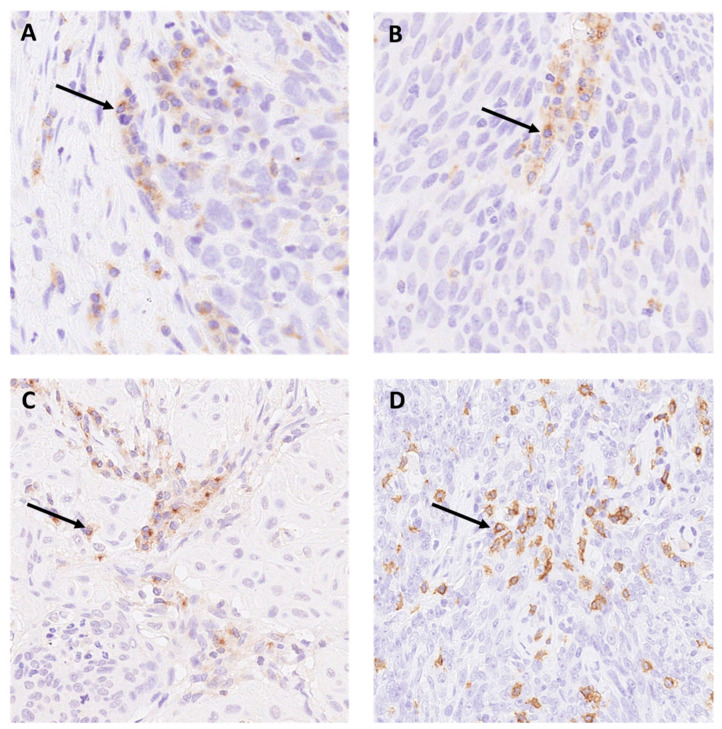
Expression of (**A**) LAG-3 on immune cells (magnification 200×). (**B**) TIM-3 (magnification 200×) (**C**) VISTA (magnification 200×) on immune cells. (**D**) Membrane-pattern of CD8 positive lymphocytes in oropharyngeal squamous cell carcinoma (OPSCC) (magnification 200×). Arrows pointing to positive staining.

Illustrative images of the staining patterns of LAG-3, TIM-3, and VISTA on immune cells are displayed in Figure 1A–D.

**Figure 2:**
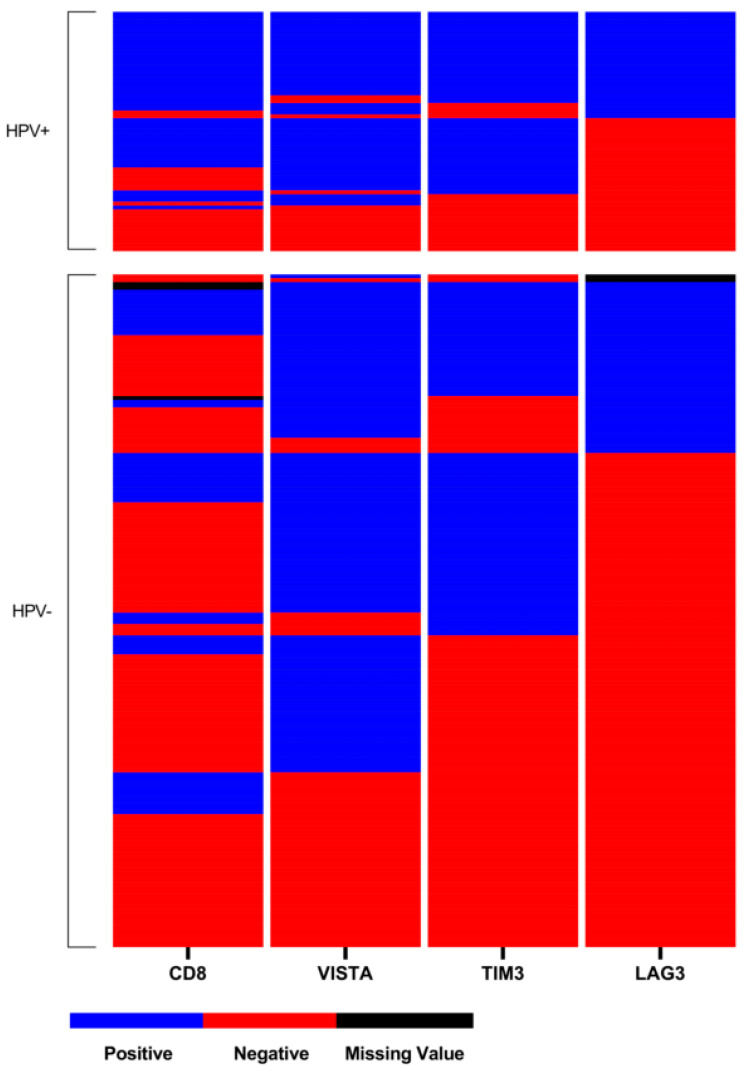
Heat-map of LAG-3, TIM-3, VISTA, and CD8 distribution within the tissue microarray (TMA). Each line presents one patient, whereas one column presents the expression of each immune-checkpoint marker on the TMA with blue indicating positive expression (>1%), red for negative expression (≤1%), and black for missing values.

There was a significant association between positive HPV-status and LAG-3 and TIM-3 expression on TILs (*p* = 0.006, *p* < 0.001; Table 2), but not for VISTA expression (*p* = 0.313; Table 2). For LAG-3 44% (*n* = 28), TIM-3 70% (*n* = 44) and VISTA 75% (*n* = 47) of HPV-related OPSCC stained positive, while only 26% (*n* = 45), 44% (*n* = 78) and 68% (*n* = 120) and of HPV-negative tumors did, respectively (Table 2). Expression of all checkpoint markers significantly correlated with each other in the entire cohort and according to HPV-status (Table 2). Composition of the expression level of ICP in OPSCC is displayed in a heat-map according to HPV-status (Figure 2). In HPV-related OPSCC, double or triple expression of ICP in association with CD8+ TILs was more frequent than in HPV-negative OPSCC (Figure 2)

A high number of CD8+ TILs was significantly associated with positive HPV-status (67% vs. 26%, *p* < 0.001; Table 2). 37% (*n* = 89) of OPSCC presented with high numbers of CD8+ TILs in their tumor microenvironment (TME) and this was significantly correlated with LAG-3, TIM-3 and VISTA expression in the whole cohort (*p* < 0.001, *p* < 0.001, *p* = 0.007).

No staining of tumor cells was observed according to LAG-3, TIM-3, or VISTA.

LAG-3, TIM-3 and VISTA expression on immune cells was associated with an inflamed tumor microenvironment (CD8+ TILs) in the entire cohort (*p* < 0.001, *p* < 0.001, *p* = 0.007,) and in HPV-related OPSCC (*p* = 0.001, *p* < 0.001, *p* < 0.001). In HPV-negative OPSCC patients, only TIM-3 expression was associated with high infiltrate of CD8+ TILs (*p* = 0.006) whereas LAG-3 and VISTA expression were not (*p* = 0.267, *p* = 0.695; Table 2).

### 2.3. Survival Analysis

Patients with HPV-related OPSCC had a significantly improved survival compared to patients with HPV-negative OPSCC (HR 0.276., CI: 0.161–0.472; *p* < 0.001). Further, LAG-3 expression (HR 0.668, CI: 0.456–0.976, *p* = 0.037), TIM-3 expression (HR 0.515, HR 0.364–0.729, *p* < 0.001), VISTA expression (HR 0.707, CI 0.500–1.000, *p* = 0.050), CD8+ TILs (HR 0.308, CI: 0.202–0.470, *p* < 0.001), younger age (HR 1.794, CI: 1.297–2.517; *p* = 0.001), low ECOG (HR 2.529, CI: 1.773–3.606, *p* < 0.001) and low UICC 7 stage (HR 0.657, CI: 0.463–0.931, *p* = 0.018) were factors for an improved OS in univariate analysis (Table 3). Multivariate analysis identified a high number of CD8+ TILs (HR 0.432, CI 0.272–0.685, *p* < 0.001), HPV-status (HR 0.430, CI: 0.244–0.757, *p* = 0.003), age (HR 1.663, CI: 1.171–2.362, *p* = 0.004), ECOG (HR 2.377, CI: 1.626–3..475, *p* < 0.001) and UICC 7 stages (HR 0.812, CI: 0.673–0.979, *p* = 0.029) to be independent factors contributing to an improved survival in the whole cohort (Table 3).

## 3. Discussion

PD-L1 expression status as a biomarker to select patients for anti-PD-1 immunotherapy in HNSCC is well investigated [42]. However little is known about the importance of the expression status of additional ICP in the TME of OPSCC, and especially according to HPV-status. The identification of new targetable ICP is gaining significance as subgroups of patients do not respond to anti-PD-1 immunotherapy in preliminary treatment or develop treatment resistance along the way. Possible reasons for this may be the interrelationship of multiple components in the tumor immune microenvironment, as it has been reported that the co-expression of LAG-3 with other inhibitory molecules such as TIM-3 or PD-1 induces the exhaustion of immune cells, resulting in downregulated cytokine expression [43,44]. As recent clinical trials are investigating alternative ICP receptors as LAG-3, TIM-3, and VISTA alone or in combination, knowledge of the expression status as biomarker is clinically relevant. Early clinical results have demonstrated success in dual immune blockage with LAG-3/PD-1 after developing resistance according to anti-PD-1 immunotherapy [45,46,47].

OPSCC, often caused by persistent infection with high-risk HPV, is a rising entity and subset of HNSCC [38]. In our cohort, 26% of OPSCC were related to high-risk type HPV. In this context, it must be acknowledged that this is not reflective of total incidences in Germany, as patient selection was performed according to suitability of tumor tissue. Nevertheless, the percentage does coincide with median incidences at our site. Higher expression of ICP in virus-related cancer has been reported as a sign for an immune-active TME [48], and viral oncoprotein expression has been proposed as biomarker for predicting success of ICP therapy [49,50]. Our data reveal a significant association between LAG-3, TIM-3, and VISTA expression in the entire cohort as well as according to HPV-status. HPV-related OPSCC had significantly higher expression rates of LAG-3 and TIM-3 and presented with higher numbers of CD8+ TILs, whereas no significant difference was detected for VISTA expression according to HPV-status.

An overexpression of LAG-3 on tumor-infiltrating CD8+ T cells in different tumor types has also been reported for ovarian cancer, hepatocellular carcinoma, gastric cancer, and follicular lymphoma [51,52,53,54]. A study by Panda et al. revealed considerably high LAG-3 expression in HNSCC and higher LAG-3 expression in association with positive HPV-status based on mRNA expression in the TCGA cohort [49]. Further, they reported that CD8A expression was highly correlated with LAG-3 expression [49], which in is line with our results.

In a phase I/IIa study, the anti-LAG-3 antibody BMS-986016 was applied in combination with nivolumab in patients with malignant melanoma who previously developed progressive disease on PD-1 blockage [55]. The objective response rate to combinations of LAG-3 and PD-1 blockage was 3.5-fold higher in patients with immunohistochemistry-based LAG-3 expression ≥1% vs. <1% [55]. This gives cause to think, that HPV-related OPSCC might be more susceptible to single or combined anti-LAG-3 antibody therapy than HPV-negative OPSCC patients. Several ongoing trials targeting LAG-3 are at range for different cancer types [56], including head and neck squamous cell carcinoma (NCT03625323).

To this point, nothing is known about the expression profile of TIM-3 in OPSCC, according to HPV-status. Liu et al., reported that the TIM-3 expression was significantly up-regulated in HNSCC compared to dysplasia or normal tissue [20] and preclinical investigation in in vitro mice models demonstrated that, inhibiting TIM-3 alone, insufficiently improves overall survival rates [57]. Clinical trials in humans are currently evaluating the safety profile and efficacy of TIM-3 alone (NCT03652077) and in combination with PD-1/PD-L1 in advanced solid tumors (NCT02817633).

To date, there is one clinical trial investigating safety and tolerability of an anti-VISTA monoclonal antibody (NCT02671955) in subjects with advanced solid tumors. Whereas nothing is known about VISTA expression in OPSCC [58], Wu et al. investigated the role of VISTA in oral squamous cell carcinoma (OSCC) and found that VISTA protein expression was significantly higher in OSCC compared to normal tissue. Further, VISTA was no independent predictor for prognosis, which is consistent with our results. Kondo et al. reported that blockage of VISTA increases T-cell recruitment to the TME of squamous cell carcinoma and that it efficiently converts CD8+ T-cells into functional effector cells in HNSCC [59]. Nevertheless, single blockage of VISTA was insufficient to reduce tumor growth compared to a simultaneous blockage of CTLA-4 and VISTA [59], recommending combined ICP-targeting in HNSCC.

To our knowledge, this is the first study investigating LAG-3, TIM-3, and VISTA expression in association with CD8+ TILs in a large cohort of OPSCC according to HPV-status. Clinical trials utilizing the safety and feasibility of LAG-3, TIM-3, and VISTA are currently on their way [60] and the first results are eagerly awaited.

In reference to the method chosen, it should be noted that when using TMAs, certain diagnostic limitations exist. Since only a small amount of tissue is harvested of each tumor, the morphological tumor heterogeneity or the heterogeneity of the infiltrating immune cells might be biased. Although we found that the TMA spots seem to represent tumor characteristics of oropharyngeal carcinoma, comparative studies on the reproducibility of TMA results should also be performed on full sections in the future to further validate our results. Concerning the evaluation of ICP expression, it has to be mentioned that we chose the criteria of 1% as it has been accepted in multiple clinical studies and recognized diagnostic scores. Increasing this cut-off would possibly disqualify cases that could actually benefit from appropriate ICP therapy regimes. However further investigations are necessary regarding applicable and reliable diagnostic scores for adequate therapeutic approaches.

Although not in the focus of our paper, survival analysis revealed that LAG-3, TIM-3, and VISTA have no significant impact on OS in multivariate analysis. Significance in univariate analysis is most likely attributable to the association with positive HPV-status and a high number of CD8+ TILs as these are both factors known to have a positive effect on OS.

In conclusion, the present study demonstrates that the co-expression of LAG-3, TIM-3, and VISTA is a frequent event in the TME of OPSCC, demonstrating an immune-rich phenotype. Therefore, it can be assumed that especially patients with HPV-related OPSCC might be susceptible to further ICP-therapy, alone or in combined regimes. However, the value of these ICI in OPSCC remains to be validated and further studies are mandatory to elucidate the role of expression status of LAG-3, TIM-3, and VISTA in relation to response rates and to establish reliable diagnostic scores for targeted immunotherapy concepts.

## 4. Materials and Methods

### 4.1. Patient Cohort

Patients who were diagnosed with OPSCC (C09, C10, International Classification of Diseases for Oncology (ICD-O)) and treated at the University Hospital Giessen between 2000 and 2016 and with sufficient pre-therapeutic tumor tissue samples available were included in this study. For preparation of tissue microarray (TMA) cores, formalin-fixed, paraffin-embedded (FFPE) cancer tissue with a thickness of 2–3 mm was mandatory, resulting in 241 samples suitable for the analysis. Clinicopathological features of the entire cohort and according to HPV-status are displayed in Table 1. Written informed consent was obtained from all patients and the study protocol was approved by the Ethics committee of Giessen (AZ 95/15, dated 19 October 2015).

The 7th edition of the International Union against Cancer (UICC) TNM classification [61] and the WHO criteria for squamous cell carcinomas of the oral mucosa [62] served as reference for tumor staging and histological grading.

### 4.2. p16^INK4a^ Immunohistochemistry, HPV-DNA Genotyping and Construction of Tissue Microarray

p16 immunohistochemistry and HPV-DNA genotyping as wells as construction of TMA were performed as previously described [50].

Briefly, for TMA construction FFPE cancer tissue with a thickness of a least 2–3 mm was mandatory to produce TMA cores. The cores were taken from a tumor area including tumor margins previously marked by a pathologist. A self-constructed semi-automated precision instrument was used to punch tissue cylinders with a diameter of 1.2 mm each from tumor tissue blocks. Subsequently, these tissue cylinders were embedded in empty recipient paraffin blocks to produce single spot TMAs and 4 µm sections were transferred to an adhesive coated slide system (Instrumedics Inc Hackensack, NJ, USA).

### 4.3. Immunohistochemistry

Immunohistochemical staining was performed on freshly cut 4 µ TMA slides by using a Bond Max automated system (Leica Biosystems, Wetzlar, Germany) in accordance with the manufacturer’s protocol.

The following monoclonal antibodies were used for immunohistochemistry: LAG-3: the rabbit IgG monoclonal antibody D2G40 (Cell Signaling Technology, Leiden, Netherlands; dilution 1:300); TIM-3: the rabbit monoclonal antibody D5D5R (Cell Signaling Technology, Leiden, Netherlands; dilution 1:100), VISTA: the rabbit IgG monoclonal antibody D1L2G (Cell Signaling Technology, Leiden, Netherlands; dilution 1:100); CD8: the mouse monoclonal antibody C8/144B (Dako/Agilent, Carpinteria, CA, USA; dilution 1:200).

Human tonsil tissue on each of the TMA slides served as control for staining. The data was evaluated independently by two experienced pathologists (KP and AQ). Discrepant results were resolved by consensus review.

### 4.4. Scoring of LAG-3, TIM-3, and VISTA

For LAG-3, TIM-3 and VISTA expression on immune cells <1% was defined as negative, whereas ≥1% of expression was considered positive. This evaluation strategy follows the established assessment of LAG-3 and PD-L1 conducted in clinical trials in malignant melanoma, where response rates of LAG-3- and PD-L1-blockage correlated with LAG-3/PD-L1 expression of >1% [63,64]. For TIM-3 and VISTA this cut-off has been retained.

For CD8 expression <50 lymphocytes/mm² were categorized as negative, whereas ≥50 lymphocytes/ mm² were classified as positive considering peritumoral and intratumoral distribution.

### 4.5. Statistical Analysis

Statistical analyses were performed using SPSS statistical software (IBM SPSS 25.0, Armork, NY, USA). Differences in patient and tumor characteristics as well as immunostaining were calculated using Fisher’s exact test or Pearson’s Chi-squares test as appropriate. Survival curves were plotted according to the Kaplan–Meier method and analyzed using the log-rank test. To assess significant differences in OS, Cox proportional-hazards models were used to estimate hazard ratios (HR) with a confidence interval (CI) of 95% for OS in univariate and multivariate analysis. All tests were two-sided and *p*-values ≤ 0.05 were considered significant for all tests. The heat-map was created using Graphpad Prism (Graphpad Prism 8.3.0, San Diego, CA, USA). Data was plotted via the heat map tool using a double gradient heat map.

## Figures and Tables

**Table 1:** Clinicopathological features of the OPSCC (oropharyngeal squamous cell carcinoma) patient cohort (*n* = 241 *).

Risk Factors		All		HPV-Related		HPV-Negative		*p*
		(*n* = 241)	100%	(*n* = 63)	26%	(*n* = 177)	74%	
Nicotine	never	44	18%	24	39%	20	11%	**<0.001**
	former/current	195	82%	38	61%	156	89%	
Alcohol	≤ 2 drinks/day	114	58%	50	96%	63	44%	**<0.001**
	> 2 drinks/day	82	42%	2	4%	80	56%	
Age	young (< 60 years)	118	49%	34	54%	84	47%	0.375
	old (≥ 60 years)	123	51%	29	46%	93	53%	
Gender	male	189	78%	44	70%	144	81%	0.057
	female	52	22%	19	30%	33	19%	
ECOG	healthy (0–1)	172	74%	45	78%	127	72%	0.417
	sick (2–4)	62	26%	13	22%	49	28%	
**Tumor characteristics**								
Localization	tonsil	126	53%	42	67%	83	48%	**0.012**
	other than tonsil	110	47%	21	33%	89	52%	
UICC7 stages	I−III	98	41%	24	38%	74	42%	0.562
	>III	141	59%	39	62%	101	58%	
T-stage	T1–3	190	79%	54	86%	135	77%	0.149
	T> 3	49	21%	9	14%	40	23%	
N-stage	N0	69	29%	8	13%	61	35%	**0.001**
	N+	170	71%	55	87%	114	65%	
M-stage	M0	222	95%	60	98%	161	94%	0.296 ^a^
	M > 0	11	5%	1	2%	10	6%	
Recurrence	no	209	87%	62	98%	146	82%	**<0.001 ^a^**
	yes	32	13%	1	2%	31	18%	
**Treatment**								
Upfront Surgery	Yes	175	73%	54	86%	120	68%	**0.006**
	No	66	27%	9	14%	57	32%	

*p*-values calculated by x² test (Pearson, asymptotic, two-sided) or ^a^ exact test (Fisher, two-sided), significant *p*-values (*p* ≤ 0.05) in bold; * 1 case with unknown human papillomavirus (HPV)-status.

**Table 2:** Relation of LAG-3, TIM-3 and VISTA expression in association with CD8-positive TILs according to each other in the whole cohort (*n* = 241) and according to human papillomavirus (HPV)-status (HPV-related oropharyngeal squamous cell carcinoma (OPSCC), *n* = 63, HPV-negative OPSCC, *n* = 177).

		LAG-3 Expression					TIM-3 Expression					VISTA Expression					CD8 Expression				
All		yes	(%)	no	(%)	*p*	yes	(%)	no	(%)	*p*	yes	(%)	no	(%)	*p*	yes	(%)	no	(%)	*p*
		73	31%	166	69%		122	51%	119	49%		168	70%	73	30%		89	37%	149	63%	
LAG-3 Expression	yes						54	74%	19	26%	**<0.001**	66	90%	7	10%	**<0.001**	39	56%	31	44%	**<0.001**
	no						68	41%	98	59%		101	61%	65	39%		50	30%	116	70%	
TIM-3 Expression	yes											113	93%	9	7%	**<0.001**	65	54%	55	46%	**<0.001**
	no											55	46%	64	54%		24	20%	94	80%	
VISTA Expression	yes																71	43%	94	57%	**0.007**
	no																18	25%	55	75%	
CD8 Expression	yes																				
	no																				
HPV-relation	yes	28	44%	35	56%	**0.006**	44	70%	19	30%	**<0.001**	47	75%	16	25%	0.313	42	67%	21	33%	**<0.001**
	no	45	26%	130	74%		78	44%	99	56%		120	68%	57	32%		46	26%	128	74%	
**HPV-related OPSCC**		**LAG-3 Expression**					**TIM-3 Expression**					**VISTA Expression**					**CD8 Expression**				
*n* = 63		yes	(%)	no	(%)	*p*	yes	(%)	no	(%)	*p*	yes	(%)	no	(%)	*p*	yes	(%)	no	(%)	*p*
		28	44%	35	56%		44	70%	19	30%		47	75%	16	25%		42	67%	21	33%	
LAG-3 Expression	yes						24	86%	4	14%	**0.026 ^a^**	25	89%	3	11%	**0.021 ^a^**	25	89%	3	11%	**0.001 ^a^**
	no						20	57%	15	43%		22	63%	13	37%		17	49%	18	51%	
TIM-3 Expression	yes											41	93%	3	7%	**<0.001 ^a^**	37	84%	7	16%	**<0.001 ^a^**
	no											6	32%	13	68%		5	26%	14	74%	
VISTA Expression	yes																38	81%	9	19%	**<0.001 ^a^**
	no																4	26%	12	74%	
CD8 Expression	yes																				
	no																				
**HPV-negative OPSCC**		**LAG-3 Expression**					**TIM-3 Expression**					**VISTA Expression**					**CD8 Expression**				
*n* = 177		yes	(%)	no	(%)	*p*	yes	(%)	no	(%)	*p*	yes	(%)	no	(%)	*p*	yes	(%)	no	(%)	*p*
		45	26%	130	74%		78	44%	99	56%		120	68%	57	32%		46	26%	128	74%	
LAG-3 Expression	yes						30	67%	15	33%	**0.001**	41	91%	4	9%	**<0.001 ^a^**	14	33%	28	67%	**0.267**
	no						48	40%	82	60%		78	60%	52	40%		32	25%	98	75%	
TIM-3 Expression	yes											72	92%	6	8%	**<0.001**	28	37%	48	63%	**0.006**
	no											48	48%	51	52%		18	18%	80	82%	
VISTA Expression	yes																32	27%	85	73%	0.695
	no																14	25%	43	75%	
CD8 Expression	yes																				
	no																				

*p*-values calculated by x² test (Pearson, asymptotic, two-sided) or ^a^ exact test (Fisher, two-sided), significant *p*-values (*p*≤ 0.05) in bold.

**Table 3:** Univariate and multivariate survival analysis according to risk factors and tumor characteristics in the whole cohort (*n* = 241).

			Univariate											Multivariate			
			Median Survival [Years]														
		N	OS	CI		*p*	5Y-OS	HR	CI				*p* ^a^	HR	CI		*p* ^a^
All				Lower	Upper				Lower		Upper				Lower	Upper	
LAG-3 Expression	no	166	4.822	3.657	5.768	**0.036**	51%						**0.037**				n.s.
	yes	73	7.148	4.163	10.127		60%	0.668	0.456		0.976						
TIM-3 Expression	no	119	3.545	1.309	5.781	**<0.001**	45%						**<0.001**				n.s.
	yes	122	7.551	n.a.	n.a.		63%	0.515	0.364		0.729						
VISTA Expression	no	73	3.129	0.489	5.768	**0.049**	45%						**0.050**				n.s.
	yes	168	5.323	4.257	6.390		58%	0.707	0.500		1.000						
CD8 Expression	no	149	2.490	1.189	3.792	**<0.001**	40%	0.308		0.202		0.470	**<0.001**				**<0.001**
	yes	89	n.a	n.a.	n.a.		78%							0.432	0.272	0.685	
HPV	HPV-negative	177	4.019	2.554	5.484	**<0.001**	44%						**<0.001**				**0.003**
	HPV-related	63	n.a	n.a.	n.a.		81%	0.276	0.161		0.472			0.430	0.244	0.757	
Age	young (<60 years)	118	8.600	5.445	11.755	**0.001**	63%						**0.001**				**0.004**
	old (≥60 years)	123	3.663	2.146	5.180		46%	1.794	1.297		2.517			1.663	1.171	2.362	
ECOG	healthy (0–2)	172	6.608	4.272	8.944	**<0.001**	61%						**<0.001**				**<0.001**
	sick (3–4)	62	1.668	0.970	2.367		31%	2.529	1.773		3.606			2.377	1.626	3.475	
UICC 7 stages	1–3	98	6.655	3.659	9.650	**0.017**	63%						**0.018**				**0.029**
	≥4	141	4.181	2.053	6.309		48%	0.657	0.463		0.931			0.812	0.673	0.979	

HR hazard ratios estimated by Cox proportional-hazards models; CI 95% confidence interval. *p*-values calculated by Log Rank (Mantel–Cox) test; univariate; *p* < 0.05 in bold; N/A: not applicable. ^a^ *p*-values estimated by Cox proportional-hazards models, uni- and multivariate; *p* < 0.05 in bold.

## Data Availability

The data presented in this study are available on request from the corresponding author.
